# Diseases of the Fingers : C—Contractions

**Published:** 1891-08-22

**Authors:** 


### SURGERY.

### DISEASES OF THE FINGERS.

C.—Contractions (*continued*).

4. Contraction of the Muscles.—The hand, like all other muscular parts, is subject to paralysis dependent on spinal and cerebral affections. The peripheral nerves are also sometimes the seat of neuritis. The result of loss of power in one set of muscles is usually to cause a deformity in the opposite direction. Besides this there may be actual active contraction from irritative lesions in the motor cells of the spinal cord. Little local treatment can be adopted in all such cases; the central disease must be attacked by the physician, and when this is improved the surgeon can sometimes remedy the abnormal position by stretching the contracted parts and using firm passive movement.

Writer's Cramp manifests itself by either spasm of the finger muscles, a sense of extreme weariness in the hand occasionally amounting to very severe pain or actual loss of power. Like most other "cramps," it is produced by continued exercise of one set of muscles in a somewhat abnormal position. In the early stages it can be cured by resting the right hand or by strapping the pen on to the back of the first and second fingers when writing. In severe cases the only thing is absolute rest from writing for weeks, or even months, with galvanism and massage. It is to be noticed that the presence of a small ganglion on any of the tendons of the wrist, or a teno-synovitis in the extensors will give rise to symptoms exactly like those of "writer's cramp"; it, therefore, behoves us to examine carefully for these affections in such cases.

5. Contraction of Ligaments.—The irritation of gouty concretions of urate of soda may set up enough inflammation to injure the lateral or anterior ligaments of the inter-phalangeal joints, causing considerable deformity. These would occur chiefly in the middle-aged or in the old. There is, however, a condition in which the first joint is affected and the finger, usually the fifth, bent at an angle of about 45 deg. or more, the terminal joint being straight. It is generally symmetrical, and occurs in young girls most frequently. It is constantly mistaken for Dupuytren's disease, and operated on by attempts to cut through bands of fibrous tissue. The result is always unsuccessful. In fact, the disease is much deeper, and is (as has been pointed out by Anderson and Adams) situated in the anterior fibres of the lateral ligament, between the phalangeal bones. This appears to be imperfectly developed, and as the bones grow and lengthen, the finger gradually assumes the flexed position. It is very like hammer toe, both in its pathology and appearance. In some cases there is a decided tendency in all the fingers to be flexed in the same way, but it is almost invariably most pronounced in the "little" finger. It is common in hysterical girls, and consequently sometimes treated by general remedies. It will be noticed, however, that there is no relaxation under an anæsthetic, which would occur in purely hysterical cases. It is usually seen for the first time about puberty, when the hands begin to be of use to their owner, but it is always congenital, or, at least, it begins soon after birth, if not before. The treatment is to carefully cut the constricting bands of ligament at the sides of the joint or in front. This is best done by a longitudinal cut large enough to enable the operator to see exactly what he is doing. Nothing is gained by operating subcutaneously, if only the strictest antiseptic precautions are taken. Neglect of these is highly culpable in all cases, but more especially when such all-important parts as the fingers are concerned. Splints made of wood or iron (*not* gutta-percha) should be kept on for some time after. The tendon may require stretching or even "splitting" (as indicated before).

6. Malformations of the Bones Causing Deformity.—The bases of the phalangeal bones may be enlarged from rheumatic disease, giving rise to lateral or anterior deviation of the fingers. Injury to the epiphysis may also give the same result. Very rarely the bones are naturally badly developed, but when this is the case it is not generally diagnosed until childhood or early youth. Little, if anything, can be done.

7. Contractions Due to Diseases or Injuries to the Nerves have been incidentally mentioned. More important, surgically, are those cases where a nerve is injured or cut through and the "antagonistic" muscles flex or extend the fingers. In all such cases an attempt should be made to reach the divided nerve and suture the ends together. Direct union of a divided nerve is not at all likely to occur, for it must be impossible, with the most exact apposition, to place the divided fibres end to end. Researches on the regeneration of nerves have been made by M. Vanlair (*Archives de biologie*, 1885), and in Germany by Teuscher, Hochwart, &c. These observers, by direct experiment, have found that the return of function after the division and re-uniting of a motor nerve is a question of months, although there is a cicatricial union of the trunk in a few days. The peripheral portion always appears to undergo rapid degeneration; first in the white substance of Schwann, and then in the axis cylinders. At the end of the fourth week there is no attempt at new growth, but soon after this the proximal end of the severed axis cylinder becomes bulbous, and sends out branches into the degenerated distal portion of the nerve. These small fibres grow rapidly, and the use of the nerve is ultimately restored by them. These investigations are of great practical importance, because they show (1) that the success of an operation for suturing the cut ends of the ulnar or median nerve must not be expected for some months; (2) that inasmuch as the degeneration after section only affects the distal portion, there is every chance of restoring the function of a nerve even after some weeks have passed since the injury. It is a common thing for patients to have loss of power or sensation in the fingers after accidents to the wrists, owing to slight injury or concussion of the nerves. If this does not improve after a few days an operation should be performed. If possible, the ends of the nerve should be brought into apposition by ligaturing the sheath or the surrounding fascia with fine catgut, avoiding actual puncture of the nerve. This cannot, however, always be effected. Splints are required for a lengthy period, but not necessarily until the regeneration has begun. So soon as the ends have firmly joined by fibrous tissue—about two weeks or so—they may be left off.

